# Qualitative exploration of perceived benefits of care and barriers influencing HIV care in trans Nzoia, Kenya

**DOI:** 10.1186/s12913-020-05236-z

**Published:** 2020-04-25

**Authors:** V. Naanyu, J. Ruff, S. Goodrich, T. Spira, M. Bateganya, C. Toroitich-Ruto, B. Otieno-Nyunya, A. M. Siika, K. Wools-Kaloustian

**Affiliations:** 1 Department of Health Policy and Management, School of Public Health, College of Health Science, Moi University, Eldoret, Kenya; 2 Department of Medicine, School of Medicine, College of Health Sciences, Moi University, Eldoret, Kenya; 3 Division of Infectious Diseases, Department of Medicine, Indiana University School of Medicine, Indianapolis, IN USA; 4 Division of Global HIV &TB, United States Centers for Disease Control & Prevention (CDC), Atlanta, GA USA; 5 Division of Global HIV &TB, CDC, Nairobi, Kenya

**Keywords:** HIV care, Barriers to HIV care, Focus group discussions, In-depth interviews, Kenya

## Abstract

**Background:**

Substantial efforts have been made to ensure people living with HIV (PLHIV) are linked to and retained in care but many challenges deter care utilization. We report perceived benefits of seeking HIV care and barriers to HIV care that were identified through a formative assessment conducted to advise the development of an alternative care model to deliver antiretroviral treatment therapy (ART) in Trans Nzoia County, Kenya.

**Methods:**

Data were collected in 2015 through key informant interviews (KIIs), in-depth interviews (IDIs), and focus group discussions (FGDs). The study involved 55 participants of whom 53% were female. Ten KIIs provided community contextual information and viewpoints on the HIV epidemic in Trans Nzoia County while 20 PLHIV (10 male and 10 female) participated in IDIs. Twenty-five individuals living with HIV participated in four FGDs - two groups for men and two for women. Key informants were purposively selected, while every third patient above 18 years at the Kitale HIV Clinic was invited to share their HIV care experience through IDIs or FGDs. Trained research assistants moderated all sessions and audio recordings were transcribed and analyzed thematically.

**Results:**

Findings showed that PLHIV in Trans Nzoia County used both conventional and complementary alternative care for HIV; however, public health facilities were preferred. Popular perceived benefits of adopting care were relief from symptoms and the chance to live longer. Benefits of care uptake included weight gain, renewed energy, and positive behavior change. Individual-level barriers to HIV care included lack of money and food, use of alternative care, negative side effects of ART, denial, and disclosure difficulties. At the community level, stigma, limited social support for conventional HIV treatment, and poor means of transport were reported. The health system barriers were limited supplies and staff, long distance to conventional HIV care, and unprofessional providers.

**Conclusions:**

Diverse individual, community and health system barriers continue to affect HIV care-seeking efforts in Kenya. Appreciation of context and lived experiences allows for development of realistic care models.

## Background

The goal of the Joint United Nations Programme on HIV/AIDS (UNAIDS) 95–95-95 strategy to end the AIDS epidemic by 2030 is that 95% of people living with HIV (PLHIV) know their status, 95% of those who are HIV positive initiate treatment, and 95% of those on treatment are virally suppressed [1]. Furthermore, there has been a concerted international effort to increase access to antiretroviral therapy (ART) for HIV-infected individuals living in low- and middle-income countries [2]. Kenya is among the high-burden countries in sub-Saharan Africa with nearly 1.5 million PLHIV. Despite efforts to achieve UNAIDS targets, by 2018 only 75% of all PLHIV in Kenya were on treatment and 63% were virally suppressed [3].

Barriers to HIV care have been well researched and can be broken down into individual, community, and health systems issues, with overlap across all three areas [4]. Individual factors frequently cited include stigma or fear of disclosure [2, 5–8], limited knowledge about HIV and its care [7, 9–11], concurrent disease, (especially drug abuse) [5, 7, 11, 12], and inadequate financial support [2, 5–7, 11, 13]. Religious and cultural beliefs [5, 7, 9, 13], negative side effects of drugs [2, 5–7, 11], lack of motivation to adhere to treatment [5, 11, 14], insufficient food support [5, 11, 15], work demands [5, 7, 11–13], and lack of insurance [12, 16] have also been cited as significant challenges to individual HIV care [9, 12]. Community obstacles include social prompting towards herbal and alternative care, limited social support [5, 7, 11] and unreliable or non-existent transportation [5, 12]. Health systems barriers in initiating and maintaining care compound these individual and community barriers to HIV care. Health system barriers consist of structural, process, and relational issues. Shortages in healthcare personnel [12, 14, 17], long distance to health facilities [4, 11, 15, 18] and poor interactions with patients are commonly reported barriers to HIV care [5, 11, 12, 14]. Other health systems barriers have included frequent appointments [11, 19, 20], unfriendly health staff with a breakdown of patient-provider trust [5, 7, 11, 14], and poor reimbursement for services by government and other financial support programs [9, 14, 16, 17].

The high attrition rates identified in PLHIV on ART in sub-Saharan Africa (SSA) make maintaining viral suppression (the last step in the cascade) even more complex [21–23]. A meta-analysis of data from HIV-care programs in resource-constrained settings estimated one and two-year attrition rates to be 22.6% (range 7–45%) and 25% (11–32%), respectively [12, 13, 24, 25]. The main reasons for attrition were death (41%) and loss to follow-up (LTFU) (59%). However, patients identified as LTFU may be either dead, in care elsewhere, or disengaged from care [12, 13, 25]. Patients disengaged from care are at high risk of morbidity, mortality, and HIV transmission. Effective strategies to keep PLHIV in care are critical to ending the HIV epidemic in SSA [10, 26–28].

As part of the development of an alternative, non-clinic-based ART delivery model, formative qualitative assessments were undertaken to advise its structure. We conducted a qualitative study in Trans Nzoia, Kenya designed to collect data on how HIV infection and HIV care were viewed and to identify barriers to care specific to this community. The community-delivered model of care (ART Co-ops) was being designed to overcome some of the barriers identified in Trans Nzoia, Kenya and ART literature. This paper describes perceived benefits of seeking HIV care and barriers to HIV care reported in the aforementioned formative qualitative assessment.

## Methods

A qualitative approach using in-depth interviews (IDIs) and focus group discussions (FGDs) was used to collect data to inform the development and implementation of the ART Co-ops model in Trans Nzoia, Kenya. This approach facilitated triangulation and convergence of data from various sources and participant categories in order to develop an understanding of perceptions of HIV care and barriers to care in Trans Nzoia communities. The data was collected between October and December 2015.

### Characteristics of study participants

All participants resided in Trans-Nzoia County, one of 47 counties in Kenya with an area of approximately 2500 km^2^ and a population > 990,000 people in western Kenya. Within the county, approximately 65% of workers are self-employed and the average monthly income is $69–$193 [29]. The county’s largest city, Kitale, is the site of the Kitale Academic Model Providing Access to Healthcare (AMPATH) Clinic which is located within the Kitale District Hospital. The AMPATH clinic supports the Trans-Nzoia catchment area providing care and treatment services to more than 19,000 PLWHIV. At the AMPATH health facility, clinic visits, ART, opportunistic infection (OI) prophylaxis and OI treatment are provided free to patients.

A total of 55 participants were enrolled across 34 data collection sessions (Table 1). Fifty-three percent of the participants were female (*n* = 29). Ten key informants from five sub-counties in Trans Nzoia were interviewed, including two peer leaders, two indigenous/traditional healers, two village chiefs, two village elders, and two religious leaders. Twenty PLHIV successfully completed IDIs. An additional twenty-five PLHIV participated in four FGDs.

**Table 1:** Study participants by methodology and site: Qualitative analysis from the ART Co-ops study in Trans Nzoia, Kenya

	**Source/site**	**# of participants**
**Key Informants**		
Peer Leaders	Kitale Kwanza	1 1
Healers	Kiminini Endebess	1 1
Chiefs	Kiminini Kwanza	1 1
Village Elders	Saboti Endebess	1 1
Religious Leaders	Saboti Kwanza	1 1
**In-depth Interviews**		
Male patients with HIV	Kitale HIV clinic	10
Female patients with HIV	Kitale HIV clinic	10
**Focus Group Discussions**		
Male patients with HIV (2 sessions)	Kitale HIV clinic	11
Female patients with HIV (2 sessions)	Kitale HIV clinic	14
**Total**		**55**

### Recruitment of study participants

Key informants were purposively recruited from each of the five sub-counties in Trans Nzoia County (Cherangany, Kwanza, Kiminini, Endebess, and Saboti) that ranged from less than 5 km, to over 20 km away from the Kitale HIV clinic. The key informants were identified by the AMPATH local community liaisons and were purposively selected based on their extensive experiences around HIV matters and awareness of the community of interest. It was determined a priori that ten interviews with the key informants would result in saturated data on the domains of interest because they were identified as having extensive depth and breadth of HIV care in the Trans Nzoia region, and there were representatives from each of the five sub counties. Once identified, key informants were invited to participate in the study and provide a range of viewpoints on HIV in Trans Nzoia.

Participants for the IDIs with PLHIV and FGDs were selected from PLHIV already receiving care at the AMPATH Kitale HIV clinic. On the day of data collection, every third adult patient presenting at the clinic was invited to participate. Twenty PLHIV (10 male and 10 female) participated in IDIs and another 25 PLHIV participated in four FGDs. Two of the groups were for men and two for women. Prior to all data collection sessions, all participants gave informed consent and were given a copy of the informed consent form to take home.

### Study tools

Session facilitators first set groups rules and norms including agreement to confidentiality. Following confirmation of continued study participation, the session facilitators used open-ended, semi-structured question guides in English and Kiswahili to ensure issues of interest were adequately discussed and for standardization across all discussion sessions (Appendix 1, 2, 3, supplementary files). The domains in the FGD and KII question guides covered common health conditions in the community, perception on the importance of HIV, local beliefs on HIV, HIV care options available, common care seeking behavior, what the community considered appropriate care response to HIV infection, sources of HIV information and health promotion in the community, and stigma associated with HIV infection.

The patient IDI tool contained all the items in the FGD and KII question guides. In addition, these IDI participants were asked about specific HIV care plans that they engaged in and factors that influenced their care journey. They were also asked to reflect on the last time they were sick and the sources of advice and care they utilized. Findings of the qualitative study helped inform the development of an alternative care model to deliver ART in Trans Nzoia County, Kenya. The present analysis focuses specifically on perceived benefits of and barriers to HIV care in the study setting.

### Data collection

Five (3 male, 2 female) trained research assistants facilitated all data collection. All were fluent in English and Kiswahili and were trained on the study, especially the eligibility criteria and consent process. They were also trained on how to run data collection sessions, data entry, transcription and coding. They had all worked in earlier qualitative projects as research assistants and were familiar with the study area. Upon obtaining informed consent from participants, facilitators for FGD first established groups rules and norms including agreement not to share information discussed outside the session.

Each FGD had a session moderator and a scribe. The male/female moderators were gender matched to sessions. All FGDs were carried out at the Kitale Hospital in a private space. One interviewer facilitated each of the IDIs which were carried out in a convenient venue selected by the interviewees. All sessions took an average of 1 h. The sessions were documented through both audio recordings and written notes. Audio recordings were translated as necessary (where English was not used) and transcribed for review and analysis.

### Data analysis

Four research assistants and one investigator independently coded the transcripts. Data were coded according to a priori, and emergent ideas and then further categorized according to thematic groupings. Where coding discrepancies occurred, all coders checked the transcripts afresh until agreement was achieved among them all. Finally, interpretative analysis involving comprehension of the context, themes, and links between themes was done. We summarize perceived benefits of seeking HIV care and barriers (individual/patient, community, and health system barriers) influencing HIV care in the study area. Illustrative excerpts are extracted from the transcripts to highlight findings.

### Ethics approval and consent to participate

The study was reviewed according to the Centers for Disease Control and Prevention (CDC) human research protection procedures and was determined to be research, but CDC was not engaged. It was also approved by the Indiana University Institutional Review Board and the Moi University College of Health Sciences/Moi Teaching and Referral Hospital University Institutional Research and Ethics Committee. Informed consent was obtained in written format from all study participants.

## Results

### Healthcare facilities utilized and perceived benefits

Conventional public and private healthcare as well as alternative care were all considered appropriate healthcare options for HIV infection. However, upon probing participants for information on the actual care utilized, public facilities were reported as the popular care option in the region.

Perceived benefits of seeking care for HIV were numerous including symptom relief, the chance to live longer, relief from stress, and boosted appetite. Other additional benefits were: weight gain, renewed energy, positive behavior change, use of prevention of mother-to-child transmission services, and being role models to fellow community members battling HIV/AIDS.

### Barriers to HIV care in trans Nzoia County, Kenya

Several obstacles to HIV care uptake were reported by key informants and PLHIV (Table 2). We categorize them into individual, community, and health system barriers.

**Table 2:** Common barriers to HIV Care: Qualitative analysis from the ART. Co-ops study in Trans Nzoia, Kenya

**Category**	**Specific barrier**
**Individual-level barriers**	Lack of money
	Disclosure difficulties
	Denial of HIV status and chronicity
	Negative side effect of drugs
	Use of alternative care
	Lack of food
**Broader community-level barriers**	Stigma
	Social prompting for alternative care
	Bad weather
	Poor means of transport
	Poverty
**Health system barriers**	Delays in service delivery
	Long distance to the conventional HIV Care facility
	Non-availability of some drugs
	Limited resources and personnel at health facilities
	Unprofessional providers

#### Individual-level barriers to HIV care

Poverty and associated lack of money was a pervasive obstacle to accessing routine HIV care. Lack of money for transportation to conventional HIV care sites and for basic subsistence were reported.

*“If you don’t have anything, you will suffer … it is like that … Yes, if you can get transport you can go to hospital to be assisted, if not, you just stay at home … especially here, lack of money and poverty is the real issue … Some people can’t afford, they live on small parcels of land so they go for manual work and get money for subsistence.” (KII, Village Elder)*

*“There are those cases where one might have been asked to visit the HIV clinic on a particular date but they postpone their visit due to lack of transport. Once they get fare, they come...” (KII, Peer 2)*

*“Money is a problem, like now I have been stopped from work and I have a very large family. My work just stopped suddenly - they started sourcing for materials from South Africa. So the problem of money is a big issue to me. Like today I have come on a bicycle from Kwanza, so transportation to clinic is becoming a challenge.” (IDI, Male Patient)*

HIV patients were reported as having difficulties disclosing their HIV status to others. They feared social stigma, which could consequently interfere with timely support from others when seeking HIV care.

*“We are trying to educate the people who have been infected to be open and disclose their condition. There are some homes where they have not accepted to stay with those people [HIV patients] - some people are under stigma … ” (KII, Peer 2)*

*“They keep it to themselves. They don’t want to disclose anything because they take the infection as something that brings shame or view it as a curse.” (KII, Religious Leader).*

Some patients initially chose to use herbal medicine, while others were in denial about the chronic nature of HIV infection. This finding was consistent with beliefs expressed around causes of HIV, which included both biomedical and indigenous explanatory models of illness.

*“They go to the witchdoctors or the church … Yes, for prayers and they use the church healing for long until when their health deteriorates. That is when they begin to seek medical attention (IDI, Male Patient)*

*“Mostly they use herbal care. There is a woman who walks around in the community collecting leaves and selling them as medicine. There is also another man who does that. Their herbs have been working like ARVs in reducing the severity. Yes, but to say someone took them and became healed of HIV? That is not true.” (KII, Religious Leader)*

Unpleasant side effects of ART medication influenced adherence to HIV care. Participants wished there was a way to minimize the discomfort experienced by ART users.

*“Another challenge for the medication, is that you are using them for a long period and you suffer from side effects … you feel some pain on your body a little bit. I don’t know if they have a remedy for that because this is the medication you are supposed to use.” (FGD, Male 2)*

*“Side effects of these drugs are really bad; you find many giving up because of that.” (FGD 1, Female)*

Participants reported limited food supplies and poor nutrition as a barrier to use of ART. Poverty was associated with the lack of resources needed to facilitate access to adequate nutrition required of a patient taking ART medication.

*“A clinic visit date may come and you don’t have even a shilling...there comes a month also in Trans Nzoia when there is drought - no food and you don’t have money and you are supposed to go to the clinic. That is a challenge.”(IDI, Female patient)*

*“ … . You know these drugs are powerful so you have to eat well before you take because they give us around three or four types of drugs....Food is a major problem to adherence because you must eat well so that the drugs can work better.” (FGD, Male 1)*

#### Community-level barriers to HIV care

Several barriers to HIV care and treatment related to the broader social and community aspects. Stigmatization of PLHIV and limited social support made the HIV care journey difficult.

*“ … Someone looks down upon me because I have HIV, if you go for example for care near home, the people in the village you meet after that will talk saying, ‘This one was in the hospital queuing with HIV people.’ Others get angry so one would rather go for treatment far from the village and pick their drugs without anyone knowing where you have come from... I didn’t want to be insulted by others … so I preferred to go far … my own parents don’t know that I’m using the drugs - the first thing you meet will be insults about being HIV infected despite the fact that I’m not sick physically, so I can’t tell them.” (IDI, Female patient)*

Lack of patient escorts especially for young patients and social influence towards use of alternative care were also reported. For instance, when some fell ill for the first time, they were advised to seek herbal and/or spiritual healing. It was only when they deteriorated that they were encouraged to take up care at the conventional HIV care facility.

*“ … There are herb healers … that’s the problem we have within the community - they still follow ways from the past. However, they are very few … In our community there is a group who think there is witchcraft, so they go there and are given herbal. They only come to the health facility when they get reduced immunity … ” (KII, Peer Leader 2)*

*“First, a woman told me look for traditional medicine - that it would heal me. So I went to the ‘reserve’ [the rural area]. While there, I became sick and almost died, in fact, I was brought to the hospital unconscious … I was completely gone...” (IDI, Male patient)*

Generally, high poverty among residents of Trans Nzoia dissuaded family members from giving continued support to HIV-infected individuals. They would facilitate care of the sick but upon realizing the costs required for the chronic disease, their support dwindled. This discouraged continued care.

*“So they [family members] take their time to help people with HIV, but when they realize that a lot of money is required, they begin to talk and say, ‘It is a waste of resources.’” (IDI, Male).*

Further, general poor infrastructure such as poor mode of transport made access to HIV care difficult. It was especially of concern during the rainy weather.

*“Sometimes you can’t find vehicles for transport. You may have the money for fare but the vehicle comes late hours... So you have to time the vehicle’s schedule because they come and go back at a given time. If you miss it, it will be a problem. Sometimes you have to skip your appointment because you missed the vehicle.” (FGD, Male 2)*

#### Health system barriers to HIV care

A number of health service delivery barriers were discussed in this study. There were reports about delays in service delivery, and long distance to conventional HIV care. This discouraged visits to the HIV clinic.

*“It’s now 1 pm and we came very early, we sit on the bench for a very long time the doctor comes and goes, I don’t know whether they are too busy … We sit on the bench for a very long time.” (IDI, Female Patient)*

*“From home to Kocholia was far, … So transportation was a challenge.” (IDI, Male Patient)*

Participants discussed non-availability of some drugs and this discouraged utilization of care. During stock outs, they would be asked to go and buy the missing drugs from pharmacies in their communities.

*“There was a time I came - my neck was stiff. I was told they didn’t have the drug I needed so I was to go and buy elsewhere.” (IDI, Female Patient)*

Other barriers noted were limited resources at the conventional HIV care facility, few staff, and some providers were said to be unprofessional as they delivered care.

*“In this village, we were built a hospital but it’s now about ten years and it has not yet been completed, and there are no doctors. People in this place have to travel to Kitale town which is far.” (IDI, Community Elder 1)*

*“Some doctors are not willing to assist us or they attend to you ‘any way they want.’ So that makes you start questioning the service.” (IDI, Male patient)*

Furthermore, there were reports of clinical staff reporting late for work, not maintaining confidentiality, and others exhibited discriminatory service.

*“The greatest challenge that I face here is time, because it interferes with my work. You might come here very early but you find the doctors have not started work … they start late sometimes like at 9:30 am.” (IDI, Male patient)*

*“There are other issues like the doctors in the hospital, some of them are bogus. They know that you are infected and they go telling people about it.” (FGD, Male 2)*

*“Sometimes you go for clinics very early but because you don’t know anyone there, you end up being examined last; other people are coming after you and they are served while you wait just because you don’t know someone to help you get served faster.” (FGD 1, Female)*

## Discussion

This study explored barriers to HIV care in order to inform the development of an alternative care model to deliver ART (ART Co-ops model) in Trans Nzoia County, Kenya. The new model was a community-based ART delivery approach established on a peer group system of PLHIV and facilitated by community health workers (CHWs). Findings showed preferred HIV care options in the area included public, private, and complementary healing. Similar studies have shown that multiple medical systems are used in the treatment of HIV in communities in Africa [10, 15, 27]. However, public facilities were reported as most popular in Trans Nzoia. It was therefore considered to be worthwhile to invest in the community-based ART model (Co-ops Model) as an extension of the public care system in the region.

The obstacles to HIV care have been previously reported as individual, community, and health system factors. On an individual level, we found that our patients faced common barriers to HIV care such as poverty, inadequate food supplies, and denial of HIV status and confirms the barriers identified by several authors including Merten’s meta-ethnography [5, 11, 12, 15, 30]. Initial use of alternative care providers as an individual obstacle to conventional HIV care in our population has been discussed before [5, 7, 11]. At the community level, social stigma, limited social support, and poor means of transport continue to affect HIV care [5, 7, 12]. Given that many of our patients walk or take uncovered transportation such as motorcycles to healthcare facilities, it was not a surprise that bad weather was also noted as a barrier to care.

Health system barriers noted in this study such as limited and unprofessional providers at healthcare facilities, and long travel distance to care have been identified in other research [12, 14, 15, 18]. This study reported some communities were up to 20 km away from the health facilities. This was likely to discourage routine care utilization at the said facilities. Non-availability of drugs was also a barrier for our patient population [6, 7].

These findings have implications for creating realistic models for HIV care in Trans Nzoia that take into account context and lived experiences. Despite advances in healthcare services, significant barriers remain in accessing HIV care [15, 27, 31]. The prevalence of HIV-related stigma and indigenous beliefs in the region indicates that continued efforts are needed to improve the education of all community members on HIV and AIDS to improve access to healthcare for PLHIV and to reduce attrition rates [11, 32]. The community-delivered care model of care (ART Co-ops) was designed to overcome some of the barriers identified in Trans Nzoia, Kenya and ART literature. For instance, individual barriers of lack of transport money and other expenses, and disclosure problems would be reduced by the new peer-based care model used in ART Co-ops. Community-level barriers of stigma, limited social support, bad weather, and unreliable means of transport would likewise diminish once the community-based model was established. Furthermore, the health system barriers – such long queues and delays in service delivery at the health facility, long distance to care, and encounters with unfriendly hospital procedures and staff – would all be curtailed with the new model.

This study has several strengths and limitations. It highlights that appreciation of localized contexts and lived experiences of PLHIV can potentially inform care programming. The study sample allowed wide perspectives to be considered. Participants in IDIs, FGDs, and KIIs were recruited from five areas of the county. This sample allowed for sharing of experiences and stories from diverse parts of the region. Generalizability of specific examples and results may not be applicable outside of Trans Nzoia County, Kenya as they are time-bound stories and descriptions of very specific individuals in a specific locale. However, the emergent themes of barriers to care including those seen at the individual, community and health system level are likely be applicable across resource constrained contexts in sub-Saharan Africa. Additional studies at other sites would help increase the generalizability of these results to other populations.

## Conclusion

Diverse barriers continue to affect care-seeking efforts among PLHIV in Kenya. An appreciation of context and lived experiences allows development of realistic care models to better serve these populations. Our study found barriers affecting utilization of HIV care in the Trans Nzoia region. The ART Co-op model was an intervention developed based on findings from ART literature and this qualitative study. It was to address some of these barriers through decreased transportation cost/time, increased social support, reduced trips to the health facility clinic, and reduced exposure to unfriendly health services. Additional research should explore how well interventions aimed at individual and environmental factors reduce perceived barriers to starting and continuing HIV care and support appropriate follow-up treatment.

## Supplementary information

**Additional file 1.**

**Additional file 2.**

**Additional file 3.**

## Data Availability

The data used in this study can easily reveal some of the informants due to the nature of the study sample and their input. To avoid breach of confidentiality, data are available from the corresponding author on reasonable request.

## Abbreviations

AIDS: Acquired immunodeficiency syndrome
AMPATH: Academic Model Providing Access to Healthcare
ART: Antiretroviral therapy
ART CO-OPS: Community delivered ART care model
CDC: The Centers for Disease Control and Prevention
CHW: Community health worker
FGD: Focus group discussion
HIV: Human immunodeficiency virus
IDI: In-depth interview
KII: Key informant interview
LTFU: Loss to follow-up
OI: Opportunistic infection
PLHIV: People living with HIV
SSA: Sub Saharan Africa
UNAIDS: United Nations Programme on HIV/AIDS
