# Supplementary material for: Qualitative exploration of perceived benefits of care and barriers influencing HIV care in trans Nzoia, Kenya
Source: BMC Health Serv Res. 2020 Apr 25;20:355. doi: 10.1186/s12913-020-05236-z (PMC7183649; doi:10.1186/s12913-020-05236-z)
Supplement: Supplementary file 1 — Additional file 1. [file 12913_2020_5236_MOESM1_ESM.pdf]

Appendix 1

ART Co-ops Study, Kenya

Key Informant Interviews

Date: ________________________
Participant's Gender: □ Male □ Female
Participant's Age: ______
Participants Community Role: _________________________________

Question Guide: In-depth interviews with key informant members of the community.

Questions are in bold with suggested prompts in italics.

1. What are some of the key health conditions in your community?
Probe: List all conditions mentioned then ask: How important is HIV?

2. **What are the health care options *actually* available and used in your community?**
Why do community members choose to utilize (insert each option mentioned sequentially)?
-Government hospitals
-Private hospitals
  -Faith based
  -NGO
  -Others
-Complementary and alternative medicine
  -Herbal
  -Spiritual
  -Chinese
-Others

3. **What are the beliefs your community holds on the origin of HIV?**
Are there any beliefs associated with the following?
-Curse
-Immorality
-Monkeys
-Witchcraft
-Others

4. **What types of labels or stigma are associated with HIV infection in your community?**

Are there any labels or stigma associated with the following?
-A perception of the infected as Contaminants
- Belief that the infected are immoral
- Perception that the medical care of the infected is a waste of scarce health facility resources
- Perception of the infected as consuming scarce household resources
- Belief that the infected bear curses
- Belief that the infected attract bad luck
-Fear of chronicity and death associated with HIV/AIDS
-Others

5. **What does your community consider an appropriate care response to HIV infection?**
List all options provided then ask, 'Would you please arrange the options beginning with the most popular?'
-Government hospitals
-Private hospitals
-Complementary and alternative medicine
-Others
