# Supplementary material for: Qualitative exploration of perceived benefits of care and barriers influencing HIV care in trans Nzoia, Kenya
Source: BMC Health Serv Res. 2020 Apr 25;20:355. doi: 10.1186/s12913-020-05236-z (PMC7183649; doi:10.1186/s12913-020-05236-z)
Supplement: Supplementary file 2 — Additional file 2. [file 12913_2020_5236_MOESM2_ESM.pdf]

Appendix 2

ART Co-ops Study, Kenya

Individual Care Seeking Behavior Interview

**Date: ________________________**<br>**Participant's Gender: □ Male □ Female**<br>**Participant's Age: ______**

Question Guide: In-depth interviews with men and women living with HIV.

Questions are in bold with suggested prompts in italics.

A. Introductory questions

1. What are some of the key health conditions in your community?
Probe: List all conditions then arrange them in order of important

2. How important is HIV?
Probes: Do people in your community believe that HIV is common?
Do you know many people in your community affected by HIV?

B. Understanding local beliefs on HIV and care options in the patient's community.

1) What are the beliefs your community holds on the origin of HIV?
Are there any beliefs associated with the following?
-Curse
-Immorality
-Monkeys
-Witchcraft
-Others

2) What does your community consider an appropriate care response to HIV infection?
List all options provided then ask, 'Would you please arrange the options beginning with the most popular?'
-Government hospitals
-Private hospitals
-Complementary and alternative medicine
-Others

C. Understanding community labeling

1) What types of labels or stigma are associated with HIV infection in your community?
- Are there any labels or stigma associated with the following?
- A perception of the infected as Contaminants
- Belief that the infected are immoral
- Perception that the medical care of the infected is a waste of scarce health facility resources
- Perception of the infected as consuming scarce household resources
- Belief that the infected bear curses
- Belief that the infected attract bad luck
-Fear of chronicity and death associated with HIV/AIDS
-Other

D. Understanding the patient's personal care seeking journey since HIV diagnosis.

Please share with me your care seeking journey.

1) When you realized that you were infected with HIV, what initial care considerations came to mind?
-Government hospitals
-Private hospitals
-Herbal care
-Spiritual healing
-Others

2) What was your assessment of the seriousness of being HIV-infected?
-Did you think it was severe?
-Did you think it was minor?

3) What specific HIV care plan/s did you ultimately engage in? Why did you select those?

4) What factors or conditions did you think would facilitate adoption of your selected care plan/s?
-What factors seemed to inhibit your plans?

5) What were the perceived benefits of adopting that particular care behavior (see answers to question 3 above)?
-Symptom relief
-Avoid death
-Others

6) Do you remember what made you seek out care? Did something happen or did someone say something to you that made you look for care?
(*Cues to action or triggers; influences promoting the care behavior)*
-Influence of friends
-Influence of family members
-Others

7) What did you do to ensure that you sustained your care activities?
-What obstacles to adherence came your way?
-Describe each obstacle and how you dealt with it

8) What things about how your community is set up or the people who live there (*structural or social characteristics)* influences your care seeking journey?
Consider the following:
-Environmental factors
-Social factors
-Health systems factors

E. Understanding the patient's current care options and situation.

1) The last time you were sick, what did you do?
Go to:
-Government hospital
-Private hospital
-Complementary and alternative medicine
-Other

2) Who do you talk to when you have health problems?
Note all that apply and ask why that particular person is ideal:
-Family member (specify)
-Health care worker (specify)
-Friend
-Workmate
-Neighbor
-Peer
-Other

3) Who do you talk to when you have questions about HIV, your body, emotions, mental health?
Note all that apply and ask why that particular person is ideal:
-Family member (specify)
-Health care worker (specify)
-Friend
-Workmate
-Neighbor

-Peer
-Other

4) What challenges do you face when considering a trip to the HIV clinic?
Note all that apply and ask why that barrier is disturbing:
-Money
-Means of transport
-Patient aide/escort
-Distance to health facility
-Quality of health services
-Availability of resources and personnel at the health facility
- Wait time at facility
- Having to take time off work
-Other

5) Are you a member of any social groups in your community (*social networks*)?
-Perhaps a church group, school group, work group, neighbor group? Elaborate (Types, Objectives, Composition, Perception…)
-Please list all types of groups that you belong to
-Describe the specific objectives of each group listed
-What is the composition/membership of each of the groups?
-Generally, how is each of the groups provided above perceived in the community?
