# Supplementary material for: Qualitative exploration of perceived benefits of care and barriers influencing HIV care in trans Nzoia, Kenya
Source: BMC Health Serv Res. 2020 Apr 25;20:355. doi: 10.1186/s12913-020-05236-z (PMC7183649; doi:10.1186/s12913-020-05236-z)
Supplement: Supplementary file 3 — Additional file 3. [file 12913_2020_5236_MOESM3_ESM.pdf]

Appendix 3

ART Co-ops Study, Kenya

Focus Group Discussion on care seeking behavior

**Date: ________________________**<br>**Participant's Gender: □ Male □ Female**<br>**Participant's Age: ______**<br>**Focus Group Number: ______________**

Question guide: Focus group discussion with HIV-infected individuals

Questions are in bold with suggested prompts in italics.

A. Introductory questions

1. What are some of the key health conditions in your community?
Probe: List all conditions then arrange them in order of important

2. How important is HIV?
Probes: Do people in your community believe that HIV is common?
Do you know many people in your community affected by HIV?

B. Understanding the sources of HIV information and health promotion in the community

1) How do social networks for People Living with HIV/AIDS or other networks provide HIV and health information in your community?
-List all types of networks available
-Describe the specific objectives of each network/group listed
- What is the composition/membership of each of the groups provided?
-Generally, how is each of the groups provided above perceived in the community?

2) How does the general media provide HIV and health information in your community?
-List all types of media that provide health information in your community
-Describe the types of HIV information each type of media provides
-Provide any recommendations you may have on how each type of media can improve its provision of HIV information

3) How do Community Health Workers provide HIV and health information in your community?
-List all cadres of CHWs that have ever worked in your community

-Describe the types of health information that they provide
-Provide any recommendations you may have on how CHWs can facilitate provision of HIV information in your community

C. Understanding local beliefs on HIV and care options.

1) What are the beliefs your community holds on the origin of HIV?
Are there any beliefs associated with the following?
-Curse-Immorality
-Monkeys
-Witchcraft
-Others

2) What does your community consider an appropriate care response to HIV infection?
List all options provided then ask, 'Would you please arrange the options beginning with the most popular?'
-Government hospitals
-Private hospitals
-Complementary and alternative medicine
-Others

D. Understanding community labeling.

1) What types of labels or stigma are associated with HIV infection in your community?
Are there any labels or stigma associated with the following?
-A perception of the infected as Contaminants
- Belief that the infected are immoral
- Perception that the medical care of the infected is a waste of scarce health facility resources
- Perception of the infected as consuming scarce household resources
- Belief that the infected bear curses
- Belief that the infected attract bad luck
-Fear of chronicity and death associated with HIV/AIDS
-Others

E. Understanding how HIV infected individuals seek care in the community.

1) Where do they turn to for help?
-Government hospitals
-Private hospitals
  -Faith based
  -NGO
  -Others
-Complementary and alternative medicine
  -Herbal

-Spiritual
-Chinese
-Others

2) What barriers are encountered during linkage to care?
Consider the following:
-Individual patient factors
-Environmental factors
-Social factors
-Health systems factors

3) What maintenance problems are encountered?
Consider the following:
-Obstacles to adherence to medication
-Drug availability/access
-Others

4) What other contextual factors influence care uptake*?*
Consider the following:
-Environmental factors
-Social factors
-Health systems factors
